# Distal Pancreatectomy for Solid Pseudopapillary Tumor of the Pancreas: A Case Report

**DOI:** 10.7759/cureus.19880

**Published:** 2021-11-25

**Authors:** Narcisa Guimaraes, Carlos Vila Nova, Raquel Oliveira Dias, Joao Fonseca Pinho, José Valente Cecílio

**Affiliations:** 1 General Surgery, Hospital Distrital da Figueira da Foz, Figueira da Foz, PRT

**Keywords:** resection, surgical, frantz, pancreas, pseudopapillary

## Abstract

Solid pseudopapillary tumor (SPT) of the pancreas is a rare tumor, sometimes asymptomatic, mainly affecting young females. It has a low malignant potential, and its complete surgical resection with free margins is the treatment of choice, offering an excellent prognosis. We present the case of a 43-year-old healthy female who was referred to the surgery department for a large abdominal mass found in the abdominal ultrasound. In the course of the study of this mass, an abdominal CT with fine needle aspiration (FNA) was performed, revealing a solid pseudopapillary tumor of the tail of the pancreas. A laparotomic distal pancreatectomy with total splenectomy was performed. A grade B pancreatic fistula occurred on the third postoperative day, and she was released on postoperative day 11. Histopathology study revealed a 10 cm solid pseudopapillary tumor of the pancreas, with cavities filled with hematic content and limited by a partially calcified capsule. SPT is a rare and indolent tumor. Prognosis is highly favorable after an adequate surgical resection, hence the importance of a precise preoperative diagnosis. Therefore, it is important to choose the most appropriate surgical strategy for each patient.

## Introduction

First described in 1959 by Virginia Frantz as papillary cystic tumors of the pancreas, solid pseudopapillary tumors (SPT) of the pancreas had several different names, acquiring their current classification since the revision of the WHO classification in 1996 [1,2]. It is a rare pancreatic tumor, accounting for 1%-2% of all tumors that affect this organ [2,3].

Located more frequently on the tail or body of the pancreas, it affects mostly women at a young age [4,5]. It usually presents a slow growth, with low malignant potential, and is asymptomatic in a significant percentage of cases [2], until it is detected in routine examinations. After its correct diagnosis, treatment consists of complete resection of the tumor lesion with free margins, which can be curative [6].

## Case presentation

A 43-year-old female with no comorbidities was referred to general surgery in-hospital consultation by her assistant physician for a “pancreatic mass, predominantly solid, heterogeneous, located in the tail of the pancreas, measuring 9 × 8 cm.” The patient was asymptomatic, but her father had recently died due to pancreatic cancer, which was worrying the patient. In this context, her assistant physician ordered an ultrasound. On observation, there was no palpable mass in the abdomen.

**Figure 1:**
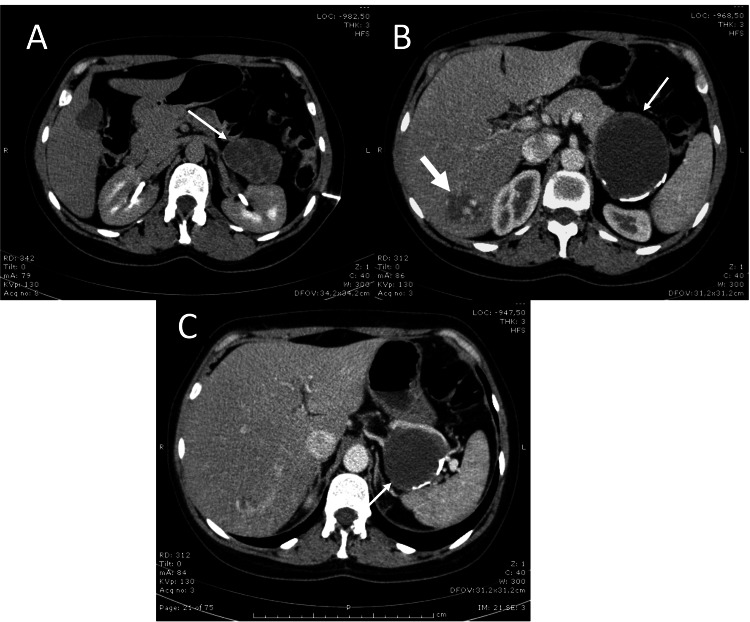
CT images of the clinical case. A lesion with solid and liquid components, delimited by a partially calcified capsule (narrow arrows), localized on the pancreatic tail; angiomatous lesion of the right hepatic lobe is also evident (bold arrow) (A, B, and C).

The in-hospital abdominal CT revealed “a massive lesion on the pancreatic tail, hypovascular and a slight enhancement effect after intravenous contrast, with coarse parietal calcifications, measuring 9.2 × 8.6 cm; in the right lobe of the liver, a nodule measuring 4 cm, corresponding to an angiomatous lesion” (Figure 1). A fine needle biopsy was performed, revealing neoplasia with solid and papillary areas consisting of round and monotonous cells, with slightly clarified cytoplasm and round, uniform, hyperchromatic nuclei or open chromatin, without evidence of mitosis. The immunohistochemical study revealed cytoplasmatic positivity of neoplastic cells for vimentin, CD10, and β-catenin. Cytokeratin CAM 5.2 and the neuroendocrine markers chromogranin A and synaptophysin were negative.

**Figure 2:**
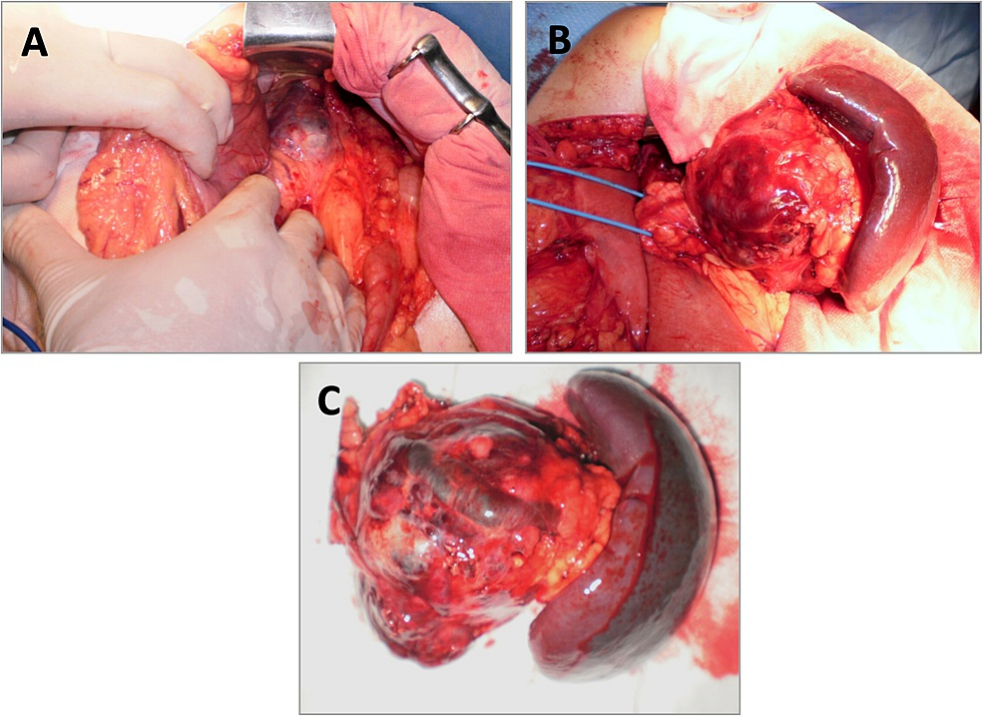
Intraoperative (A and B) and specimen (C) images showing the tumor on the pancreatic tail, without cleavage plane with the splenic vessels.

A laparotomic distal pancreatectomy with splenectomy was performed. Splenic preservation was not attempted since there was no apparent cleavage plane between the tumor and the splenic vessels (Figure 2).

In the postoperative period, the patient developed a grade B pancreatic fistula detected on postoperative day 3, and subcutaneous octreotide was initiated. On postoperative day 5, an abdominal CT was performed, revealing the presence of a liquid collection in the splenic locus, measuring 9 × 9 cm, which was percutaneously drained. The patient was initiated on enteral diet on the sixth postoperative day and was discharged on the 11th day.

The patient was periodically observed in the general surgery consultation, and the fistula closed spontaneously several weeks later. The drain was removed 10 weeks after surgery.

Histopathological examination of the surgical specimen revealed a tumor lesion measuring 10 cm at the greater axis, with several cavities filled with hematic material encapsulated by a focal calcified capsule. There was an intimate relation between the lesion and the splenic vessels, without tumor infiltration. Six lymph nodes were identified, without tumor infiltration.

The patient remains asymptomatic and without signs of recurrence eight years after the surgical procedure.

## Discussion

SPT is a rare type of pancreatic tumor. It most often affects young women [2,4,5]. Patients may present with epigastric pain, nausea, vomiting, abdominal fullness, and others [2,4,5] or be asymptomatic (in up to 30%) [5,7]. In the clinical case presented, the patient was asymptomatic, and the SPT was an incidental finding.

SPT usually exhibits characteristic imaging features such as a well-circumscribed, round or lobulated lesion with clear-depicted peritumoral capsule, and depending on the degree of hemorrhagic necrosis, it may vary from a solid- to a thick-walled cyst. Intravenous contrast produces a peripheral heterogeneous enhancement [3]. Despite these characteristics, performing a needle biopsy, when possible, can help establish a preoperative diagnosis. Endoscopic ultrasound fine needle aspiration (EUS-FNA) is becoming the gold standard for diagnosis [8].

Upon histological examination, the light microscopic features are characteristic and include cells arranged in the form of solid sheets, microcysts, and pseudopapillae. The solid areas contain sheets of uniform polygonal cells admixed with a delicate vascular network, and areas of hemorrhage and necrosis are seen, secondary to the vascular network that traverses the tumor, and rarely may present peripheral, capsular calcifications [3]. Pseudopapillae are formed by central thin-walled blood vessels surrounded by several layers of dropped neoplastic cells.

Upon immunohistochemical examination, SPT usually reveals positivity for vimentin, CD10, CD56, α1-antitrypsin (AAT), and progesterone receptor (PR), as well as cytoplasmic and nuclear immunoreactivity for β-catenin. Loss of cell membrane immunoreactivity and/or nuclear immunoreactivity for E-cadherin is seen in all cases of SPT [9,10].

In our case, the abdominal CT already showed a lesion with imaging characteristics suggesting SPT, and it was decided to perform an FNA, whose histological and immunohistochemical result came to solidify the diagnosis.

The combination of clinical, imaging, histological, and immunohistochemical data is usually enough to establish a preoperative diagnosis of solid pseudopapillary tumor, enabling the surgeon to program the best surgical treatment for each patient.

The preferred treatment modality for SPT is complete tumor resection, even in the presence of local invasion or metastasis [2,5,11]. The procedure of choice depends on the location and size of the tumor, as thus the surgical approach (laparotomic versus laparoscopic). Lymph node metastasis is very rare, so extensive lymphadenectomy is not indicated [5]. In our case, the laparotomic approach was preferred over the laparoscopic approach because of the size of the tumor, as well as the possible invasion of the splenic vessels. The surgical procedure was complicated by a pancreatic fistula, which was considered the most frequent surgical complication [5]. The patient was treated with somatostatin analogs, and despite the surgical drain, the patient developed a liquid collection in the splenic locus, which we managed by (ultrasound-guided) percutaneous drainage, which classifies this fistula as a grade B pancreatic fistula [12].

The prognosis of patients diagnosed with SPT is quite favorable, with overall survival at five years higher than 95% after surgical treatment [5]. Despite its usually indolent course, SPT has malignant potential, with recurrence and/or metastasis being reported in up to 10% of patients [11].

## Conclusions

SPT is a rare tumor, which affects mainly young women. In these patients, SPT should always be considered in the differential diagnosis of a solid or partially cystic lesion located in the pancreas or upper abdomen. Its imaging characteristics can strongly suggest the diagnosis; in less obvious cases, a needle biopsy can consolidate the imaging diagnosis and help establish the best surgical treatment for each patient, which can be curative.
